# Periprosthetic Hip Fractures around the Stem: Can the Stem Design Affect Fracture Features?

**DOI:** 10.3390/jcm13092627

**Published:** 2024-04-29

**Authors:** Luca Costanzo Comba, Luca Gagliardi, Francesco Onorato, Fabrizio Rivera

**Affiliations:** 1 Orthopedics and Trauma Department, SS Annunziata Hospital, ASL CN1, 12038 Savigliano, Italy; 2 Orthopedics and Trauma Department, Univertità degli Studi di Torino, 10100 Turin, Italy

**Keywords:** cementless, periprosthetic femur fracture, stem design, total hip arthroplasty

## Abstract

**Background:** Total hip arthroplasty is one of the most successful orthopedic surgeries; nevertheless, many of these surgeries are the causes of failure, and among them, periprosthetic fractures are one of the major causes of revision. Our study focuses on periprosthetic hip fractures with two different stem designs. The aim of the study was to analyze the obtained results, focusing on the features of periprosthetic stem fractures observed. **Methods:** We retrospectively reviewed periprosthetic fractures occurring between 2010 and 2023, involving Alloclassic^®^ or CLS^®^ uncemented femoral stems. We analyzed demographic data, proximal femur morphology, and the fracture type. **Results:** We identified 97 patients. Considering the proximal femur morphology, we found that there was statistically significant prevalence of Dorr A proximal femur morphology in the CLS^®^ group and of Dorr C in the Alloclassic^®^ group. Considering the distribution of the fracture pattern, we reported a non-statistically significant prevalence of the fracture pattern with stable stems in the CLS^®^ group. **Conclusions:** The choice of the prosthetic design of the femoral stem is a crucial element when planning total hip arthroplasty. However, we found a non-statistically significant difference between the two stems considered, raising questions about the real role of stem design as a primary determinant of periprosthetic hip fractures.

## 1. Introduction

The ever-evolving field of orthopedics has experienced significant advancements in total hip arthroplasty (THA), including better materials, new prosthetic designs, and minimally invasive approaches. This development has allowed an improvement in functionality and quality of life for countless individuals [1]. The constantly growing number of implants suggests a potential increase in revision interventions in the future [2]. Periprosthetic femoral fractures (PFFs) remain one of the major causes of revision surgeries, with considerable implications for patients’ functional outcomes, morbidity, and mortality [3]. Moreover, they contribute to increased healthcare costs due to prolonged hospital stays, extended rehabilitation periods, and high rates of hospital readmission [4]. 

Although the literature has already suggested several patient risk factors, including age, gender, and the presence of osteoporosis and rheumatoid arthritis, the contribution of implant design on PFF risk is less clear [5]. Recent studies have suggested that cemented femoral stem fixation could reduce the risk of intra-operative and post-operative PFFs following THA for femoral neck fractures (FNFs), as well as after elective THA [6,7,8].

A cemented femoral stem leads to a modification of the load transfer in the case of both taper slip and composite beam designs [6,7]. Notwithstanding, cementless fixation remains widely performed, such as in the United States, possibly due to shorter surgery times, concerns about potential embolization risks, and the challenges associated with revision surgeries. Despite the decades of research focused on optimizing load transfer at the prosthesis–bone interface, stress shielding is still considered one of the important factors in the long-term survival of femoral stem components. For this reason, the use of patient-specific stem designs considering individual characteristics to provide optimal implant solutions is rapidly increasing [9]. Nowadays, a wide range of stem designs is currently available on the market, allowing surgeons to optimize the stem choice based on patient femur characteristics [10]. Nevertheless, there is still an ongoing debate regarding the various factors influencing the incidence and type of fractures around the stem [11].

Even though numerous classifications have been proposed, the historical Vancouver classification [12] remains widely used and reliable, primarily due to its simplicity in guiding treatment decisions and reproducibility. Moreover, understanding the intricate interplay between patient factors, surgical variables, and implant characteristics necessitates thorough investigation and careful consideration to reduce the impact of periprosthetic hip fractures. Surprisingly few reports in the literature reported the clinical PFF rates among different cementless stem designs.

In our study, we retrospectively analyzed the observed PFFs at a single institution, comparing the two different stem designs implanted with a cementless fixation technique. Given the wide array of stem designs available, the endpoint of our investigation sought to determine whether specific stem designs may be correlated with a specific fracture pattern.

## 2. Material and Methods

**Figure 1:**
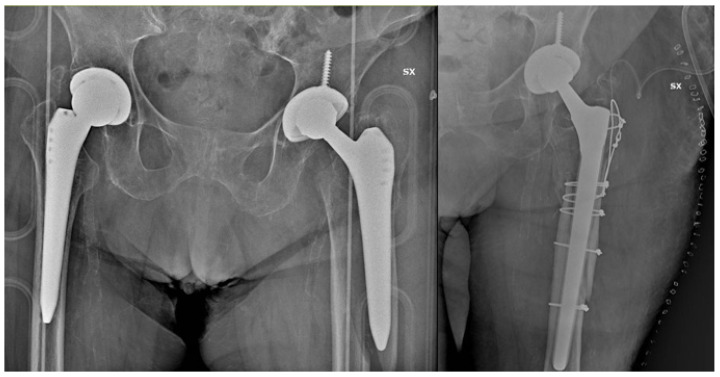
Periprosthetic fracture with unstable stem (Alloclassic), which underwent revision surgery.

**Figure 2:**
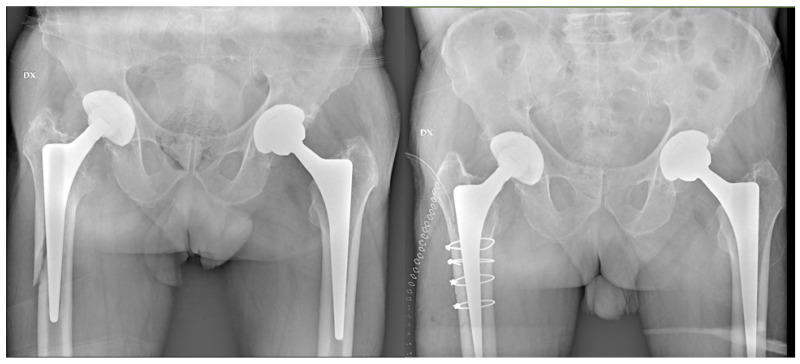
Periprosthetic fracture with stable stem (CLS), which underwent osteosynthesis surgery.

We retrospectively reviewed the periprosthetic fractures of the proximal femur which occurred in the period between 2010 and 2023 and were surgically treated at SS Annunziata Hospital, Savigliano, Italy. Among them, we focused on periprosthetic fractures involving CLS^®^ or Alloclassic^®^ Zimmer (Woso, IN, USA) uncemented femoral stems. Patients were therefore divided into two distinct groups: Group A, comprising individuals with PFFs who previously underwent uncemented primary hip replacement with the Zweymuller Alloclassic^®^ stems (Zimmer), and Group B, consisting of patients with PFFs on a Spotorno CLS^®^ stem (Zimmer) [Figure 1 and Figure 2].

We only considered PFFs following primary THAs originally implanted for hip osteoarthritis (OA). Only post-operative fractures occurring at least one month after the surgery were included, therefore excluding all intra-operative and early post-operative fractures within the first month after surgery (to minimize the risk of including intra-operative fractures not seen during surgery). Additionally, we excluded THAs on previous failed osteosynthesis and pathological fractures. We only selected patients who underwent a complete preoperative radiological study, including an antero-posterior (AP) and axial X-ray of the pelvis and hip as standardized in our institution; for the most complex cases where stem loosening was uncertain, a preoperative CT study was also performed. 

Cases were analyzed according to demographic data (gender and age), proximal femur morphology through the Dorr classification (due to the interobserver and intraobserver reliability) [13,14], and fracture type conforming to Vancouver classification (because of the reproducibility and the simple clinical application) [12]. In this regard, a re-evaluation of implant stability and osteointegration was always performed intraoperatively for definitive diagnosis before proceeding with the appropriate treatment, so that an eventual change in the initial assessment could then be reported in the surgical report and correctly retrieved by the authors.

Demographic data among the two groups, as well as proximal femur morphology and the fracture pattern distribution, were compared. Statistical analysis was performed using the Fisher’s test to compare nominal variables. *p* values of <0.05 were considered to be significant.

## 3. Results

We identified 97 patients with periprosthetic fractures of the proximal femur in the examined period. Among them, 37 (38%) were male and 60 (62%) were female, with a mean age of 81 (range 51–96) years. In Group A, we reported 55 patients, of which 13 (24%) were male and 42 (76%) were female, with a mean age of 81 years (range 51–96). In Group B, we reported 42 patients, of which 24 (57%) were male and 18 (43%) were female, with a mean age of 81 years (range 65–96). No difference was found regarding the average age of patients; however, we reported a statistically significant prevalence of women in Group A (*p* = 0.0014), as depicted in Table 1.

Considering the distribution of proximal femur morphology with Dorr classification, we found 4 cases of Dorr A, 35 cases of Dorr B, and 16 cases of Dorr C in Group A; conversely, we found 18 cases of Dorr A, 21 cases of Dorr B, and 3 cases of Dorr C in Group B [Table 2]. Comparing these results, we found that there was a statistically significant prevalence of the Dorr A proximal femur morphology in Group B (*p* = 0.000095) and a statistically significant prevalence of the Dorr C proximal femur morphology in Group A (*p* = 0.009).

Considering the distribution of fracture patterns with Vancouver classification, we found 6 cases of type A fractures, 19 cases of B1, 17 cases of B2, 9 of B3, and 4 type C fractures in Group A. Conversely, we found 4 cases of type A fractures, 21 of B1, 12 of B2, 5 of B3, and no cases of type C PFFs in Group B, as reported in Table 3. It could be inferred that the femoral stem was assessed as stable in 54 cases (56%) and unstable in 43 cases (44%). Fracture patterns with stable stems had a higher incidence in Group B (60% vs. 53%); thus, the unstable pattern was more common in Group A. However, this difference was evaluated as not statistically significant (*p* = 0.5415).

## 4. Discussion

The main finding of the present study was a significant incidence of PFFs in Dorr A femoral phenotypes which underwent THA with the Spotorno CLS^®^ (Zimmer) femoral stem. In contrast, patients with a Dorr C femoral bone were subjected to PFFs in a statistically higher proportion when implanted with the Zweymuller Alloclassic^®^ stems (Zimmer). However, no significant difference in a specific fracture pattern occurrence or stem loosening was observed between the two stems.

The great success of THA and the large number of patients undergoing this procedure over the years clearly highlight the importance of managing complications as a priority. Among these complications, PFFs play an important role, and even more so within an audience of increasingly elderly patients [15]. In fact, periprosthetic fracture represents the fourth cause of failure of a THA, after aseptic loosening, hip dislocation, and the wear of materials [16]. 

The selection of the femoral stem design in hip arthroplasty represents a crucial decision involving considerations such as the material, geometry, fixation method, and modularity. No prosthetic stem can perfectly replicate the physiological load transmission; each stem is associated with a specific load pattern and consequently with a specific periprosthetic bone remodeling. In 2018, Rivière and colleagues [17] analyzed long-term bone remodeling in five widely used types of femoral stems, including those evaluated in our study. They observed a meta-diaphyseal grip in the Alloclassic stem, with proximal cortex atrophy as a primary sign of stress shielding, typically appearing approximately two years post-implantation, but stabilizing at around five years.

Conversely, the CLS stem’s triple taper tended to achieve both metaphyseal and metaphyseal-diaphyseal grip, generating compressive forces with proximal load transfer, thereby partially mitigating bone remodeling secondary to stress shielding, especially in proximal femurs with a Dorr A type morphology. Conversely, in cases of Dorr C type morphology, the presence of signs of stress shielding indicated osteointegration at the inter-subtrochanteric level.

While the role of cementation in preventing PFFs (defined by the interplay of implant load and stiffness on one side, and bone or compound material resistance with bone cement on the other) seems clear in the literature, with stem fixation considered one of the most important revision risk factors in older patients [18,19], for younger patients preserving optimal bone stock, the influence of prosthetic stem design on the type of PFFs is less defined [5]. 

In the present study, the Zweymuller stem was implanted more often in Dorr type C “stovepipe” femoral shafts with a relative greater risk of PFF. This finding could be interpreted in two different ways. The underlying femoral osteopenia typical of Dorr type C, also confirmed by Dorr’s original histological studies, could demand the use of a cemented stem, irrespective of the patient’s age. On the other hand, the findings might be a selective bias related to surgeon stem choice at the time of surgery, where achieving a solid diaphyseal fixation in type C proximal femur diaphyseal-engaging stems was the preferred choice.

However, recent studies of modern femoral stem designs have demonstrated durable fixation with the use of cementless fixation in Dorr type C bone, although this was only performed by highly experienced surgeons [20,21]. A recent study by Jeong, Sang-Jin, et al. [22] found that a taper rectangular stem (i.e., an Alloclassic stem) was associated with a higher incidence of PFFs and femoral stem revision compared to flat taper and quadrangular taper stems. We know that in a non-cemented implant, femoral stems with different designs lead to different load transmission at the level of the prosthesis–bone interface, favoring phenomena such as stress shielding and bone resorption. Whenever an accidental fall occurs, this different stress distribution may thus influence a different location of the fractures [23]. This phenomenon has also been studied in cemented implants. In 2020, Windell and colleagues [24] compared three different polish tapered cemented stems by recreating periprosthetic fractures in sawbone models and observing their biomechanics, location and the energy needed to reach the breaking point.

Within our cohort, we reported a significantly higher prevalence of women with PFFs in Group A compared to Group B; this finding is consistent with several studies in the literature reporting a higher risk of PFFs in females. Konow, Tobias, et al. identified several PFF risk factors by analyzing over 200,000 cases from the German Arthroplasty Registry, including elderly patients, females, and uncemented and collarless stem designs [25]. Similarly, in 2021, Sershon and colleagues [26] attributed greater influence to the stem design in determining the risk of PFFs within three months post-surgery compared to other factors, such as the surgical approach choice, and reported a 2.6-fold and a 2.3-fold increased risk of PFFs with collarless or single-wedged stems compared to collared or fit-and-fill stems, respectively.

In a 2019 study [27], the incidence and pattern of periprosthetic fractures were analyzed based on anatomical or straight stem designs, and the authors reported a higher overall incidence of periprosthetic fractures with anatomical stems, which was also associated with a higher incidence of Clamshell-type fractures compared to Vancouver type B fractures. Matthias Luger et al. investigated the rate of PFFs within the first year post-surgery between cementless short and straight stem THA, concluding that short stem THA reduces Vancouver type A PFFs in the trochanteric region compared to straight stem THA, while Vancouver type B fractures are comparable [28].

In our study, we found that in cases of a periprosthetic hip fracture in the presence of an Alloclassic stem, there is a relative greater percentage of stem loosening compared to that found in the presence of a CLS stem; however, this difference was not statistically significant. 

Although the choice of stem during the initial implantation seems to be influenced by achieving metaphyseal filling for an optimal press-fit, and therefore by the Dorr index, with a statistically significant prevalence of Dorr type C femurs in the Alloclassic group compared to a prevalence of type A in the CLS group, the larger dimensions of the metaphyseal portion of the Alloclassic stem could partly influence fracture biomechanics and bone integration loss. Considering the need for different surgical treatment in cases of prosthetic stem instability in a periprosthetic fracture (i.e., stem revision and reimplantation in the case of B2 and B3 Vancouver fractures), with the associated impact on patient outcomes, if this trend is confirmed with greater statistical power, it will introduce another factor to consider when choosing the initial implant prosthetic stem. This will be particularly important in Dorr B-type femoral canals, where both prosthetic models can ensure an adequate press-fit.

We encourage the use of preoperative templating to ensure a stem choice which fits the geometry of a patient’s femoral canal to decrease the risk of iatrogenic fractures in patients who undergo THA. Lastly, we must consider the elderly age of this patient population and their associated comorbidities, which in some cases may have led the surgeon to opt for synthesis even if revision would have been indicated, in order to limit surgical time and associated intra- and post-operative complications [29].

Our study has several limitations. Firstly, the retrospective design and limited sample size. Moreover, the heterogeneity of the two groups in terms of the female ratio represents another confounding factor; we observed a greater presence of females in Group A, which was statistically significant, and therefore we cannot exclude that this may have influenced our results. Additionally, the exclusion of fractures occurring intraoperatively or within the first month post-surgery to minimize the risk of including intraoperative fractures not observed during surgery might bias the study. Despite the clear literature evidence regarding modifiable and unmodifiable PFFs risk factors that may already guide surgeon preferences [30,31,32], further studies are necessary to build evidence capable of deeply influencing our clinical practice.

## 5. Conclusions

Periprosthetic hip fractures represent a challenging complication in the realm of orthopedic surgery, particularly around the femoral stem, prompting a critical examination of the potential influence of stem design on fracture features. When approaching a first implant, the choice of prosthetic design of the femoral stem must be carefully taken into consideration to achieve a good press-fit in relation to the morphology of the proximal femur. In our study, the Alloclassic stem reported a relatively higher incidence of stem instability in cases of periprosthetic fracture compared to that detected for the CLS stem, although with a non-statistically significant difference. On the other hand, the lack of a significant difference in fracture patterns between the Alloclassic and CLS stems raises questions about the real role of stem design as a primary determinant of periprosthetic hip fractures. While the biomechanical characteristics of these stems differ, it appears that other factors, such as patient demographics, bone quality, and surgical technique may exert a more substantial influence on fracture outcomes. These findings highlight the need for a more comprehensive understanding of the multiple factors contributing to periprosthetic fractures, and call for further research to refine risk stratification and improve patient outcomes in hip arthroplasty.

## Figures and Tables

**Table 1:** Patients’ demographics.

Patients, n	Alloclassic (A)	CLS (B)	*p* Value
Patients, n	55	42	
Sex (male/female), n	13/42	24/18	*p* = 0.0014
Mean age	81 (51–96)	81 (65–96)	*p* > 0.05

**Table 2:** Proximal femur morphology (Dorr classification).

	Alloclassic (A)	CLS (B)	*p* Value
Dorr A	4	18	*p* = 0.000095
Dorr B	35	21	*p* > 0.05
Dorr C	16	3	*p* = 0.009

**Table 3:** Fracture pattern (Vancouver classification). Green cells: stable stem. Red cells: unstable stem.

Vancouver	Alloclassic (A)	CLS (B)	*p* Value
A	6	4	*p* > 0.05
B1	19	21	*p* > 0.05
B2	17	12	*p* > 0.05
B3	9	5	*p* > 0.05
C	4	0	*p* > 0.05

## Data Availability

For full disclosure, a paper copy is available at SS Annunziata Hospital, Savigliano, Italy.
